# Assessing patterns of genetic admixture between sheep breeds: Case study in Algeria

**DOI:** 10.1002/ece3.3069

**Published:** 2017-07-08

**Authors:** Sahraoui Harkat, Abbes Laoun, Ibrahim Belabdi, Rédha Benali, Djouhar Outayeb, Nathalie Payet‐Duprat, Véronique Blanquet, Mohamed Lafri, Anne Da Silva

**Affiliations:** ^1^ Laboratoire des biotechnologies liées à la reproduction animale Université de Blida Blida Algeria; ^2^ Université de Djelfa Djelfa Algeria; ^3^ Ecole nationale supérieure vétérinaire d'El‐Harrach Alger Algeria; ^4^ INRA UMR1061 Génétique Moléculaire Animale Limoges France; ^5^ Université de Limoges UMR1061 Génétique Moléculaire Animale Limoges France

**Keywords:** conservation, cross‐breeding, hybridization, local breeds, microsatellites, spatial analysis

## Abstract

In developing countries, cross‐breeding between local breeds and indigene or exotic breeds represents one of the main threats to the livestock diversity, leading to genetic dilution and loss of unique allelic combination underlying essential local adaptive traits. In this study, two Algerian sheep breeds, known to be highly admixed, were considered as a case study, to demonstrate how combination of different methodologies coupled with the use of specific softwares can be efficient to assess the spatial structuration of a hybrid zone, even in a case of extreme admixture. A fine sampling covering distribution areas of both breeds was implemented in order to study the admixture area and adjacent zones from a phenotypic (i.e., 19 quantitative traits were considered) and a genetic point of view (i.e., 21 microsatellites markers were used). Both approaches gave concordant patterns, highlighting areas with sheep most differentiated (or less admixed) for each breed. In detail, the region of Biskra appeared as the most preserved for the Ouled‐Djellal breed and the northwest of Laghouat was identified as the most preserved area for the Rembi breed. The approach proposed in the study offers a low‐cost solution to identify the most representative flocks of a breed, allowing the implementation of efficient conservation plans.

## 1 INTRODUCTION

Many areas of biology focus on the phenomenon of hybridization, that is, the interbreeding between individuals from genetically distinct populations resulting in at least some offspring of mixed ancestry (Barton & Hewitt, 1989; Futuyma & Shapiro, 1995). Given the particularity of the processes occurring in hybrid zones, these areas are of considerable interest from an evolutionary point of view; such as Hewitt (1988) defined them as “natural laboratories for evolutionary studies.” In conservation biology, hybridization between native and “exotic” stocks is of great concern. Indeed, the original genetic structure can be considerably impacted by the genetic admixture, inducing the loss of variants adapted to local conditions and jeopardizing the integrity and viability of local populations (Olden, Leroy, Douglas, Douglas, & Fausch, 2004; Rhymer & Simberloff, 1996). Until then, the main goal of studies focusing on hybrid zones was to assess admixture between wild native populations and wild exotic populations or domesticated populations (e.g., Biosa et al., 2015; Harvey, Glover, Taylor, Creer, & Carvalho, 2016; Muñoz‐Fuentes, Vilà, Green, Negro, & Sorenson, 2007; Oliveira et al., 2015). To our knowledge, the problem has never been investigated considering only domesticated populations in spite of the importance of this issue in the livestock conservation field.

According to FAO, across the world, one‐third of farm animal breeds face extinction, and cross‐breeding with exotic breeds usually exported from developed to developing countries or with indigenous breeds, represents one of the greatest threat for livestock diversity, particularly in developing countries (FAO 2014, 2015). It is precisely in developing countries that the greatest genetic diversity can be recorded (Da Silva & Benjelloun, 2016). Breeds of these areas are still largely managed in a traditional way (i.e., with management practices characterized by “soft” artificial selection and important place for natural selection (Taberlet et al., 2008)) and then have remained closely connected to their original habitat (Hoffmann, 2013) unlike “industrial” breeds that are submitted to harsh artificial selection. Cross‐breeding leads to the homogenization of the livestock genetic pool and induces a genetic dilution of the specific allelic combinations, selected over millennia by natural and artificial selection that confer to the valuable local breeds strong adaptation to their environments.

According to FAO, across the world, one‐third of farm animal breeds face extinction, and cross‐breeding with exotic breeds usually exported from developed to developing countries or with indigenous breeds, represents one of the greatest threat for livestock diversity, particularly in developing countries (FAO 2014, 2015). It is precisely in developing countries that the greatest genetic diversity can be recorded (Da Silva & Benjelloun, 2016). Breeds of these areas are still largely managed in a traditional way (i.e., with management practices characterized by “soft” artificial selection and important place for natural selection (Taberlet et al., 2008)) and then have remained closely connected to their original habitat (Hoffmann, 2013) unlike “industrial” breeds that are submitted to harsh artificial selection. Cross‐breeding leads to the homogenization of the livestock genetic pool and induces a genetic dilution of the specific allelic combinations, selected over millennia by natural and artificial selection that confer to the valuable local breeds strong adaptation to their environments.

In Algeria, Ouled‐Djellal, one native sheep breed considered more productive, is favored by most of the breeders. Algerian farmers under increasing economic pressure realize unsupervised cross‐breeding (i.e., not carried out in the framework of selection plans) between Ouled‐Djellal and local Algerian breeds, with the hope to improve their productivity (Madani, Yakhlef, & Abbache, 2003). The practice is so extensive that three breeds have been found highly genetically admixed with the Ouled‐Djellal: Rembi, Taâdmit, Berber and to a lesser extent Barbarine (Gaouar, Da Silva, et al., 2015; Gaouar, Kdidi, & Ouragh, 2016). The situation between Ouled‐Djellal and Rembi appeared as the most critical: Gaouar, Da Silva et al. (2015), by use of 30 microsatellite markers, recorded a pairwise F_ST_ of 0.009 [0.002–0.017]_95%_ and by genome‐wide single‐nucleotide polymorphism genotyping, obtained a value for the pairwise F_ST_ not significantly different from zero (*p*‐value = .001) between the breeds.

In this study, we considered the extreme case of admixture between Ouled‐Djellal and Rembi as a case study, in order to analyze at a fine geographic scale, the hybrid zone, from a phenotypic and genetic point of view. The study was conducted in the Algerian region where the most part of Ouled‐Djellal and Rembi are encountered, that is, including three zones, the cradles of each breed and the contact zone intermediate between them. Microsatellites markers were used to infer genetic diversity. Our objectives were to assess spatial distribution of genetic admixture in the different zones and to determine in which genetic and phenotypic patterns overlapped. This study demonstrated how combination of different methodologies (Wright's F statistics (1951), discriminant analyses of principal components, spatial analyses of principal components) coupled with the use of softwares specially designed to identify and estimate individual and/or population admixture (FLOCK (Duchesne & Turgeon, 2012), NEWHYBRIDS (Anderson & Thompson, 2002)) can be efficient to dissect the spatial structuration of the hybrid zone, whereas in case of extreme admixture classical approaches are ineffective; giving consequently strong foundation to the development of conservative plans.

## 2 MATERIALS AND METHODS

### 2.1 Sampling design

Sheep were sampled in their respective distribution areas (i.e., areas where they are mostly encountered): Ouled‐Djellal was sampled in Biskra, M'Sila, Djelfa and in the south of Laghouat; Rembi was sampled in Tiaret, Djelfa and in the north of Laghouat, covering a surface area of around 105,854 km^2^. Tiaret region (considered as the cradle of the Rembi breed) is dominated by Rembi, whereas Biskra (considered as the cradle of the Ouled‐Djellal breed) and M'Sila areas are dominated by Ouled‐Djellal; the middle area, including Djelfa and Laghouat, represents the overlapping zone supposed to favor strongest admixture (see Figures 2 and 3). The sampling area was largely located between the Saharan Atlas and the high Tellian plateaus. Overall, 54 farms were visited during spring 2013 in order to phenotype sheep of both breeds, and 78 farms were visited during spring 2014 in order to collect blood samples.

#### 2.1.1 Phenotype sampling

A total of 1,793 sheep were phenotyped: 1,098 Ouled‐Djellal (distributed as follows by regions: Djelfa 364, Laghouat 356, M'Sila 180, Biskra 198) and 695 Rembi (distributed as follows by regions: Djelfa 273, Tiaret 320 and Laghouat 102) were considered (see details of GPS coordinates Table S1). For each individual, 19 quantitative traits were assessed: body weight, head length, head width, ear length, ear width, neck length, body length, chest girth, cannon perimeter, tail length, trunk length, chest width, internal width of the chest, external width of the chest, chest depth, rump length, hip width, ischium width, withers height (for details considering the process of phenotyping see Harkat et al., 2015 and Laoun et al., 2015).

#### 2.1.2 Blood sampling

A total of 152 individuals blood samples were collected with vacutainer tubes which have EDTA as preservative. Ninety‐two animals from Ouled‐Djellal (21 from Djelfa, 22 from Laghouat, 20 from M'Sila and 29 from Biskra) and 60 animals from Rembi (19 from Djelfa, 20 from Tiaret and 21 from Laghouat) were sampled (see details of GPS coordinates in Table S1).

### 2.2 DNA extraction, polymerase chain reaction (PCR), and fragment analysis

DNA was extracted from the blood samples according to the salting out procedure (Miller, Dykes, & Polesky, 1988). Twenty‐three microsatellites were amplified; all the microsatellites except CSSM66 and INRA35 were chosen through the panel of microsatellites proposed for sheep characterization by the Food and Agriculture Organization of the United Nations/International Society for Animal Genetics (FAO/ISAG) (2011). All the details concerning conditions of PCR amplification are available in Othman et al. (2016). The amplified PCR products were loaded on an ABI 3730 Genetic Analyzer using LIZ‐600 as internal size standard (Applied Biosystems). Amplified fragment lengths were assigned to allelic sizes with GeneMapper v.4.0 (Applied Biosystems).

### 2.3 Data analysis

The mean number of alleles per breed, the average observed (H_o_) and expected (H_e_) heterozygosity over loci per breed were estimated using ARLEQUIN 3.5 (Excoffier & Lischer, 2010). To calculate allelic richness and the richness of private alleles, we used the rarefaction method (Kalinowski, 2004) implemented in HP‐RARE (Kalinowski, 2005) adopting a sample of 44 individuals. Polymorphic information content (PIC) and effective number of alleles (Na_e_) were estimated for all markers using the Molkin software (version 2.0) (Gutiérrez, Royo, Alvarez, & Goyache, 2005).

Departures from Hardy–Weinberg equilibrium (HWE) and linkage disequilibrium (LD) among loci were estimated using the program GENEPOP v4.0 (Rousset, 2008). Levels of significance were adjusted using the false discovery rate (FDR) procedure (Benjamini & Hochberg, 1995).

Frequencies of null alleles at each locus and for each breed were estimated with INEST (http://genetyka.ukw.edu.pl/INEst10_setup.exe), in order to take into account simultaneously null allele frequencies at each locus and the average level of the intrapopulation inbreeding as a multilocus parameter (Chybicki & Burczyk, 2009).

The unbiased estimator of Wright inbreeding coefficient, F_IS_, was calculated following Weir and Cockerham (1984) (f estimator). Its significance was assessed using a permutation method (10,000 permutations) implemented in the GDA (Lewis & Zaykin, 1999).

The extent of population subdivision was examined by calculating the global multilocus F_ST_ value. The index of pairwise F_ST_ of Weir and Cockerham (1984) and their associated 95% confidence intervals were determined using GDA (Lewis & Zaykin, 1999).

To assess the degree to which groups of individuals differ from each other, from a genetic and a phenotypic point of view, we performed discriminant analyses of principal components (DAPC), using the approach implemented in the ADEGENET package (Jombart, 2008) within the statistical package R version 3.2.2 (R Core Team 2015). For the “phenotypic” DAPC analysis, quantitative variables were size‐corrected using the residuals after regression on overall body size.

We conducted spatial analyses: (1) spatial analysis of principal components (sPCA), a multivariate method optimizing the identification of spatial genetic patterns, was performed with the package ADEGENET (Jombart, 2008) using as connection network, the Delaunay triangulation (Upton & Fingleton, 1985); (2) a similar approach was conducted considering phenotypic traits by the use of MULTISPATI‐PCA (Dray, Said, & Debias, 2008), that takes into account the spatial location of samples. Computations were conducted with the “ade4” (Chessel, Dufour, & Thioulouse, 2004) and “spdep” packages (Bivand, Pebesma, & Gomez‐Rubio, 2008) for the R statistical software package. For both analyses, Monte Carlo tests were used to check the statistical significance of the spatial structures (global and/or local spatial structure) for 10,000 iterations. The sPCA results were visualized by plotting the samples according to their geographic coordinates and coloring them according to their respective scores along the first sPCA components.

The approach of Duchesne and Turgeon (2012) implemented in the software FLOCK was also used to estimate the level of genetic differentiation between groups of sheep. FLOCK is a non‐Bayesian method, specially designed to provide spatial and/or temporal admixture maps. This method provides high resolution power even when most genotypes are admixed.

Finally, The NEWHYBRIDS software v1.1 Beta (Anderson, 2008; Anderson & Thompson, 2002) was used to identify most preserved individuals from admixture. We ran the software with the following parameters: a burn‐in period of 20,000 generations and 200,000 MCMC, “Jeffery's like priors,” and using three genealogical classes that correspond to: (1) “Pure” Ouled‐Djellal, (2) “Pure” Rembi, and (3) admixed individuals modeled as F2 hybrids.

## 3 RESULTS AND DISCUSSION

The current study investigated, for the first time, an area of admixture between two sheep breeds at fine scale, with the principal objective of identifying most preserved flocks of each breed. Ouled‐Djellal and Rembi, local sheep breeds of Algeria, were considered as a case study of critical admixture analysis, and the flocks were evaluated from phenotypic and genetic point of view.

### 3.1 Genetic diversity

The twenty‐three microsatellites loci were highly polymorphic (see details in Table S2); a total of 293 different alleles (mean = 12.70 per locus ± 3.51) were found in the two breeds, effective number of alleles ranged from 3.02 for MAF209 to 8.27 for MAF70 (mean = 5.15 per locus ± 1.73,) with a mean PIC of 75.78 ± 8.40. On average, 95.80% of individuals were successfully typed for each microsatellite (the worst score was found for DYMS1 with 86.18% of successful genotypes obtained).

The software INEST suggested possible null alleles for more than 20% of individuals, for two microsatellites (HUJ616 and MAF65); these observations were made in both breeds. Hence, we decided to exclude these microsatellites from the following analyses.

Considering both breeds, the average observed heterozygosity (H_o_) was 0.77 ± 0.10 and average expected heterozygosity H_e_ was 0.78 ± 0.08. All the genetic diversity indices considered were found really close between the two breeds (Table 1). F_IS_ values were low which was consistent with the fact that no microsatellite was found at Hardy–Weinberg disequilibrium after correction for false discovery rate, in both breeds (Table 1). The mean F_IS_ found was not significantly different from zero (F_IS_ = 0.05 [−0.002; 0.113] IC_95%_)_._

**Table 1:** Genetic diversity measured by breed

Breed	*n*	MNA (*SD*)	R (PR)	Ho (*SD*)	He (*SD*)	Loci not in HWE (FDR*^3^)	F_IS_ IC_95%_
Ouled‐Djellal	92	11.71 (3.06)	10.41 (1.44)	0.76 (0.09)	0.78 (0.08)	3 (0)	0.03 [0.06;0.01]
Rembi	60	11.00 (2.98)	10.36 (1.39)	0.79 (0.11)	0.78 (0.07)	3 (0)	−0.01 [−0.05;0.02]

*n*, sample size; MNA, mean number of alleles; *SD*, standard deviation; R, allelic richness; PR, private allelic richness; He, expected heterozygosity; Ho, observed heterozygosity; HWE, Hardy–Weinberg equilibrium; FDR*3, loci not in HWE after False Discovery Rate correction.

We failed to detect significant linkage disequilibrium between pairs of loci in each considered breed after False Discovery Rate correction.

### 3.2 Spatial structuration

Considering both breeds, the mean F_ST_ was very low: 0.003 [0.001; 0.005] (IC_95%)._ Pairwise F_ST_ values calculated between breeds of the different administrative regions (Laghouat/M'Sila/Biskra/Tiaret/Djelfa) were also very low; however, four pairwise F_ST_ values were found significantly different from zero (close to 0.01). In details, Ouled‐Djellal sheep from Biskra appeared genetically different from sheep of Djelfa (regardless of the breed) and also from Rembi sheep of Laghouat; moreover Ouled‐Djellal sheep of Djelfa were found genetically different from Rembi sheep of Laghouat (Table 2).

**Table 2:** Pairwise F_ST_ among two Algerian breeds grouped by administrative regions (with confidence intervals at 95%)

	Djelfa O.D.	Msila O.D.	Laghouat O.D.	Biskra O.D.	Djelfa R.	Tiaret R.
Msila O.D.	0.005 [−0.002; 0.013]					
Laghouat O.D.	−0.001 [−0.007; 0.006]	−0.002 [−0.008; 0.004]				
Biskra O.D.	**0.010 [0.001; 0.018]**	0.003 [−0.004; 0.010]	0.002 [−0.004; 0.011]			
Djelfa R.	0.002 [−0.003; 0.007]	0.010 [−0.000; 0.023]	0.002 [−0.007; 0.012]	**0.013 [0.004; 0.024]**		
Tiaret R.	0.002 [−0.005; 0.010]	0.000 [−0.006; 0.008]	−0.003 [−0.010; 0.003]	0.004 [−0.004; 0.014]	0.005 [−0.005; 0.015]	
Laghouat R.	**0.013 [0.006; 0.022]**	0.006 [−0.000; 0.013]	−0.000 [−0.007; 0.006]	**0.013 [0.004; 0.026]**	0.004 [−0.003; 0.012]	0.006 [−0.002; 0.015]

O.D., Ouled‐Djellal; R., Rembi. In bold pairwise FST significantly different from zero.

**Figure 1:**
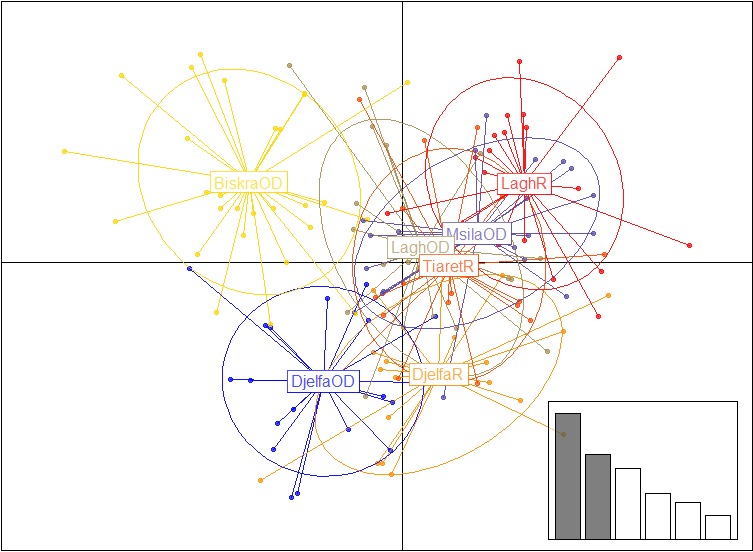
Scatterplot of the first two principal components of DAPC using breeds considered by regions and genotyped with 22 microsatellites, as prior clusters. Breeds are labeled inside their 95% inertia ellipses, and dots represent individuals. The inset indicates the eigenvalues of the first principal components. OD, Ouled‐Djellal; R, Rembi; Lagh, Laghouat

In the genetic DAPC analysis, approximately 83% of the total genetic variability was retained. On the scatterplot of the first two components of the DA (Figure 1) Ouled‐Djellal from Biskra appeared particularly distinct from sheep of Djelfa and also from Rembi of Laghouat; moreover, Ouled‐Djellal of Djelfa was particularly distinct from Rembi of Laghouat. Hence, these results gave conclusions highly correlated to those obtained below with the pairwise F_ST_ values.

**Figure 2:**
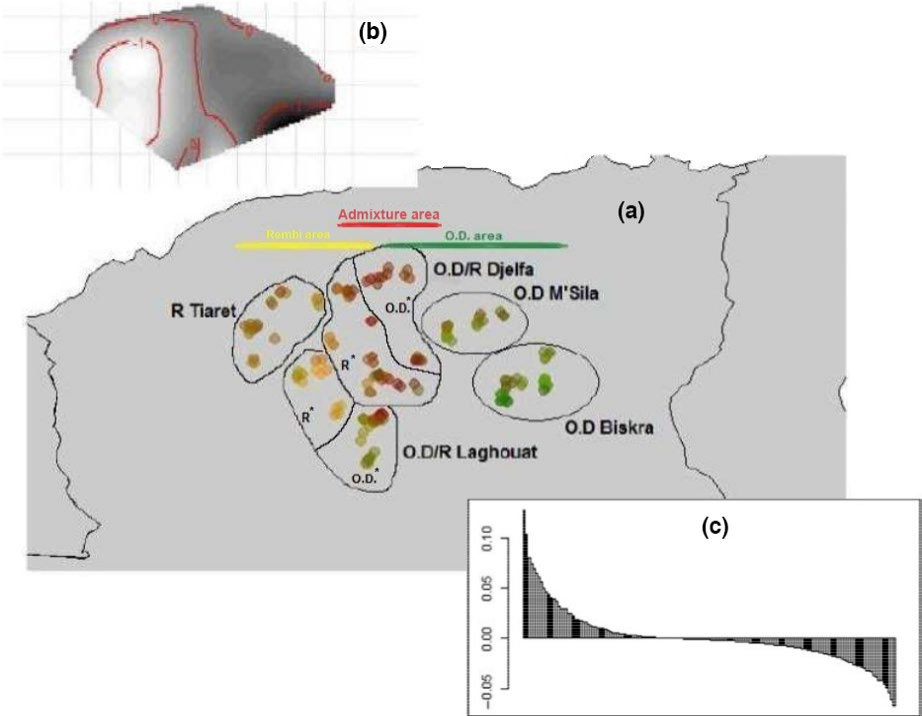
Graphical display of the first two axes of the sPCA analysis considering distribution areas of two Algerian breeds (a); interpolation of sPCA scores (b); sPCA eigenvalues for each global and local axis (c). OD, Ouled‐Djellal; R, Rembi; R* indicated that most flocks in the delimited area belong to Rembi breed; OD* indicated that most flocks in the delimited area belong to Ouled‐Djellal breed

In the phenotypic DAPC analysis, approximately 89% of the total genetic variability was retained. On the scatterplot of the first two components of the DA (Figure 2) groups of sheep were distributed according to triangular pattern; at one angle appeared Djelfa Ouled‐Djellal, the second was occupied by M'Sila Ouled‐Djellal/Biskra Ouled‐Djellal and the last was occupied by Laghouat Rembi. The essential distinction appeared, here again between Ouled‐Djellal of Biskra, Ouled‐Djellal of Djelfa and Rembi of Laghouat. Moreover, this analyse highlighted the phenotypic similarity between Ouled‐Djellal of M'Sila and Ouled‐Djellal of Biskra clustering together and separately from other groups.

**Figure 3:**
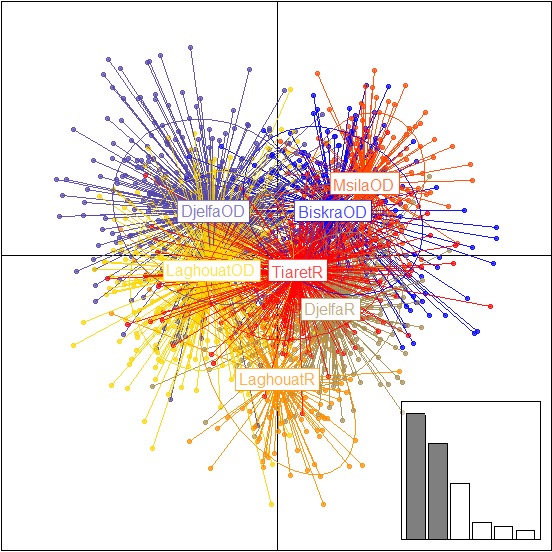
Scatterplot of the first two principal components of DAPC using breeds considered by regions and phenotyped with 19 morphometric traits, as prior clusters. Breeds are labeled inside their 95% inertia ellipses, and dots represent individuals. The inset indicates the eigenvalues of the first principal components. OD, Ouled‐Djellal; R, Rembi

The spatial PCA performed on genetic data was carried out using Delaunay triangulation as connection network with the sheep considered. Considering eigenvalues, the two‐first scores were distinguished from other eigenvalues suggesting the possibility of a spatial pattern (Figure 3c). The global Monte Carlo test (10,000 iterations) indicated no significant global spatial structure; only a trend was detected (*p*‐value = .08). The eigenvectors of the two‐first global scores were plotted to the geographic coordinates highlighting three zones with a color gradient from Biskra Ouled‐Djellal to Rembi Laghouat (i.e., the two zones identified as most dissimilar) (Figure 3a,b). In details, in the Ouled‐Djellal area, flocks of Biskra (colored in deep green) and to a lesser extent of M'Sila (colored in green) were clearly distinct from Ouled‐Djellal flocks of Djelfa (colored in red) and Ouled‐Djellal of Laghouat (appearing as a mixture between green and red flocks). In the Rembi area, the most distinct zone was represented by flocks of Laghouat (colored in yellow); they were clearly separated from flocks of Djelfa (colored mostly in red) and more discretely from flocks of Tiaret (colored in brownish‐yellow). Hence, in the overlap area supposed to be the most intense admixture area (i.e., area of Djelfa and Laghouat), Rembi and Ouled‐Djellal appeared effectively clearly admixed in Djelfa (colored in red), but the situation was quite different in Laghouat with Ouled‐Djellal of the southeast admixed with sheep of Djelfa, whereas Rembi of the northwest of Laghouat showed a clear genetic particularity.

**Figure 4:**
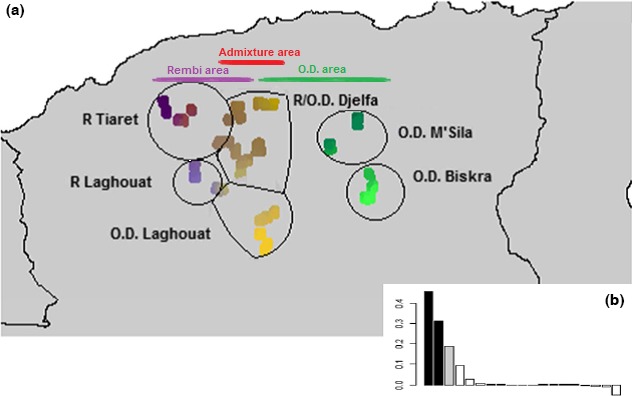
Graphical display of the first two axes of the MULTISPATI‐PCA analysis, considering distribution areas of two Algerian breeds (a); MULTISPATI‐PCA eigenvalues for each global and local axis (b)

The MULTISPATI analysis gave similar pattern including three areas: green zone of Biskra (close to M'Sila), brown‐yellow central area of Djelfa (with Rembi and Ouled‐Djellal sheep) and Laghouat Ouled‐Djellal, and finally purple area of Laghouat Rembi close to Tiaret Rembi area, which however contained few brown‐yellow flocks. The global Monte Carlo test (10,000 iterations) indicating significant global spatial structure (*p*‐value = .001) (Figure 4).

Given the critical level of admixture recorded in the study, classical Bayesian methods (e.g., implemented in the STRUCTURE software, Pritchard, Stephens, & Donnelly, 2000) used to infer the optimal number of clusters (*K*) were inappropriate. We then used FLOCK, with the aim to test the significance of the pattern that emerged from the previous analyses; in this perspective, samples were partitioned into three clusters (*K* = 3) using the “samples mode” to specify that initial partition should include Rembi from Laghouat and Ouled‐Djellal from Biskra in single and different clusters. For each sample, the number of allocations to each cluster was computed (Table 3). A majority of Ouled‐Djellal from Biskra was assigned to K1 (66.7%); Ouled‐Djellal from Biskra were mainly assigned to K2 (69%), with to a lesser extent Ouled‐Djellal from M'Sila. Rembi from Laghouat were mainly assigned to K3. The global *p*‐value was highly significant (*p*‐value = .0016) pointing out the existence of the genetic structure tested.

**Table 3:** Percentage of individuals of each sample allocated to a cluster, using the method implemented in FLOCK, for *K* = 3

Breed considered (administrative region)	% of individuals allocated to K1	% of individuals allocated to K2	% of individuals allocated to K3	Total number of allocated individuals
Ouled‐Djellal (Djelfa)	**66.7%**	14.3%	19.0%	21
Ouled‐Djellal (M'Sila)	23.8%	**52.4%**	23.8	21
Ouled‐Djellal (Laghouat)	33.3%	33.3%	33.4%	21
Ouled‐Djellal (Biskra)	6.9%	**69.0%**	24.1%	29
Rembi (Djelfa)	30.0%	20.0%	50.0%	20
Rembi (Tiaret)	35.0%	25.0%	40.0%	20
Rembi (Laghouat)	0.0%	25.0%	**75.0%**	20

*K*: number of cluster, in bold percentages of assignment superior to 50%.

**Figure 5:**
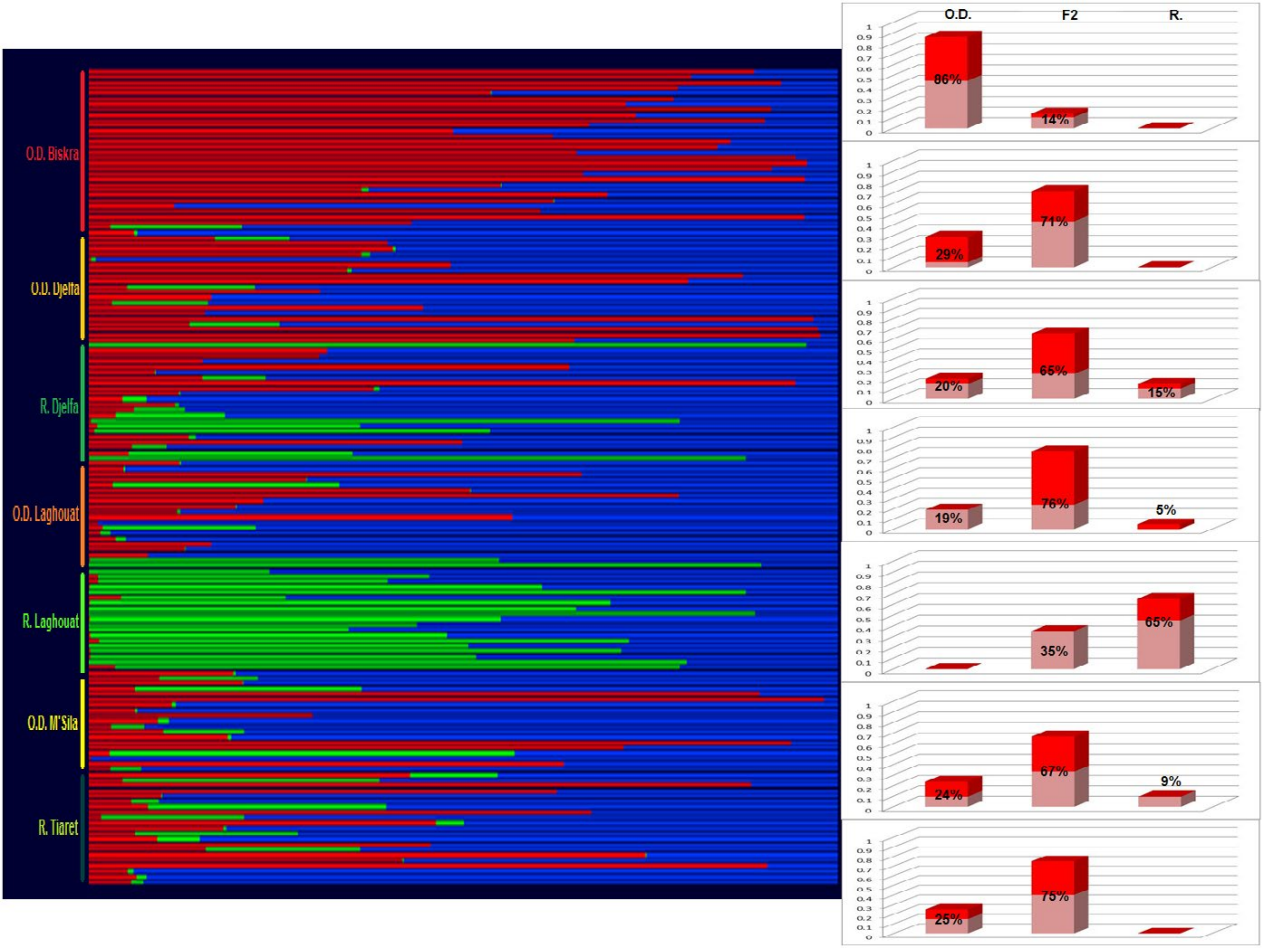
Results of NEWHYBRIDS analysis considering 153 sheep genotyped with 21 microsatellites. On the main figure, the posterior probability, qi, of individuals belonging to distinct genealogical classes is represented for each individual; the three classes considered are “pure Ouled‐Djellal” in red, “pure Rembi” in green and “F2 hybrids” in blue. On the right, histograms show the proportions for each sample, of individuals assigned to one of the class: in pink proportion of individuals assigned to a class with 0.5 < qi < 0.8, and in red individuals assigned to a class with qi > 0.8

Finally, we used the NEWHYBRIDS program to classify individuals further into genealogical classes. This analysis is a probability‐based model, which computes through MCMC, the posterior probability, qi, of individuals belonging to distinct genealogical classes. According to Anderson (2008), the software performs better when “pure” representatives of the parent breeds are specified a priori. Hence, based on previous results, we specified that Ouled‐Djellal from Biskra and Rembi from Laghouat had highest likelihood to be composed of “pure” individuals of each breed. The Figure 5 displays posterior probability for each individual to be assigned to one of the classes “pure Ouled‐Djellal,” “pure Rembi,” or “F2 hybrids.” Histograms (on the right of the Figure 5) summarize the results, by giving the proportions for each sample, of individuals assigned to one of the category (in pink proportion of individuals assigned to a class with 0.5 < qi < 0.8, and in red individuals assigned with qi > 0.8). The objective of this analysis was to determine whether “pure” individuals could be identified in other regions than Biskra (for Ouled‐Djellal) and northwest of Laghouat (for Rembi). Whereas, “pure Ouled‐Djellal” were identified in all the samples considered (excluding Laghouat Rembi) in proportions close to 20%, few individuals were found assigned to the class “pure Rembi” (out of Laghouat Rembi), and interestingly none of them was identified in Tiaret (the cradle of the Rembi breed).

The parallel and complementary approaches conducted (phenotypic and molecular genetic) gave correlated patterns and identified clearly the areas of Biskra for Ouled‐Djellal and northwest of Laghouat for Rembi as most preserved zones. Hence, (1) as expected, the cradle of Ouled‐Djellal (i.e., Biskra, where Ouled‐Djellal is still dominant) corresponded to the most preserved area for this breed; (2) Contrary to what one might expected, the cradle of Rembi (i.e., Tiaret, where Rembi is still dominant) was not identified as the most preserved area for Rembi; (3) it was precisely in the overlap zone (i.e., Djelfa and Laghouat) where admixture was supposed to be stronger, that the most preserved area for the Rembi breed was identified (northwest of Laghouat).

Rembi is subjected to intense practices of cross‐breeding with Ouled‐Djellal (MATET, 2009), considered by the breeders as the most productive breed. The situation reaches such a point that for specialists, initial specimens (characterized by uni‐colored bay‐fawn fleece, short neck and massive horns, as described by Chellig, 1992) have quite totally disappeared. This hardy breed, particularly well adapted to highlands poor pastures in the steppe, numbers 2 million heads (FAO DAD‐IS 2003, www.fao.org/dad-is) and is classified as vulnerable according to DAGRIS (www.dagris.ilri.cgiar.org). The results of this study postulated that conservation plans have to consider in priority the area including the northwest of Laghouat, as breed management of this region allowed to preserve a part of the genetic originality of the Rembi breed.

## 4 CONCLUSION

A fine scale analysis of this type implies considerable sampling effort (in order to cover the distribution ranges of the breeds); microsatellites, as cost effective‐markers, constitute an affordable solution and hence represent valuable option to infer genetic diversity in such case. This study showed that the use of microsatellite markers combined with spatial analyses (using different methodologies and specialized softwares) constitutes relevant solution to assess genetic subtle substructures, even in situation of strong admixture. Cross‐breeding is one of the main threats affecting the genetic diversity of livestock (FAO 2014, 2015). For example, only in North Africa, high levels of admixture were recently reported among Egyptian goats (Elbeltagy et al. 2016), Turkish sheep (Yilmaz, Cemal, & Karaca, 2014), Tunisian sheep (Kdidi et al., 2015), and among Moroccan sheep (Gaouar, Kdidi, et al. 2016). Locally adapted breeds of livestock represent true reservoirs of biodiversity; they are the result of unique evolutionary phenomena, showing strong adaptation to harsh environments. These highly resilient breeds, offering key solution to deal with the global warming issue, are endangered, and in the same way, the worldwide food security is threatened. The ability to analyze hybridization zones and to identify most preserved areas from genetic dilution is essential in such a context, allowing implementing conservation plan and preserving this unique genetic heritage.

## CONFLICTS OF INTEREST

None declared.
