# Supplementary material for: Assessing patterns of genetic admixture between sheep breeds: Case study in Algeria
Source: Ecol Evol. 2017 Jul 8;7(16):6404–12. doi: 10.1002/ece3.3069 (PMC5574784; doi:10.1002/ece3.3069)
Supplement: Supplementary file 1 [file ECE3-7-6404-s001.docx]

**Table S1.** Geographic localization of sheep included in the phenotypic and genetic sampling.

| **Breed name** | **Region** | **Phenotypic sampling** | **Blood sampling** |
| --- | --- | --- | --- |
| REMBI | DJELFA | - 35°27'12.75"N 2° 30'31.07"E (10) - 34°45`25.5" N 2°25`10.4" E (34) - 34°48`22.9" N 2°26`19.7" E (33) - 34°26`13.7" N 2°31`14.7" E (31) - 34°27`36.1" N 2°32`10.7" E (30) - 34°37`54.9" N 2°40`08.1" E (43) - 34°37`34.07" N 2°33`33.38"E (30) - 34°35`51.57" N 2°33`38.22"E (30) - 35°29`31.8" N 2°59`49.5" E (32)   n = 273 | - 34°23`29.5" N 2°25`10.4" E (1) - 34°45`25.5" N 2°25`10.4" E (3) - 34°11`45.7" N 3°42`1.8" E (2) - 34°13`21.6" N 3°29`40.2" E (2) - 34°18`16" N 3°07`33.2" E (3) - 34°30`14.0" N 3°01`27.7" E (2) - 35°26`51.21" N 2°31`03.85" E (2) - 35°28`35.96" N 2°59`52.98" E (2) - 34°57`12.89" N 3°03`30.60" E (2)   n = 19 |
|  | TIARET | - 35°15`04" N 2°18`01.3" E (40) - 35°17`34.6" N 2°32`50.1" E (33) - 35°14`35.5" N 1°26`05.7" E (36) - 34°55`04.7" N 2°18`31.2" E (30) - 34°55`02.9" N 2°08`43.3" E (30) - 34°54`07.6" N 2°05`57.8" E (30) - 35°31`46.1" N 1°01`50" E (31) - 35°24`30.2" N 1°35`01.3" E (30) - 35°15`29.4" N 1°19`46.9" E (30) - 35°22`47.9" N 1°05`54.4" E (30)   n = 320 | - 34°53`01.06" N 1°17`49.35"E (2) - 34°54`22.39" N 1°19`03.65"E (2) - 34°54`03.67" N 1°12`35.33" E (2) - 34°57`02.62" N 1°15`04.98" E (2) - 34°50`02.82" N 1°34`01.04" E (1) - 35°28`33.66" N 1°16`05.68" E (3) - 35°13`18.68" N 1°33`00.51" E (2) - 35°24`07.30" N 1°44`32.27" E (3) - 35°15`23.25" N 2°14`12.85" E (2) - 35°15`02.35" N 2°18`02.24" E (1)   n = 20 |
|  | LAGHOUAT | - 33°49`55.3" N 5°11`1"E (12) - 34°23`02.8" N 1°38`40.2"E (28) - 34°32`29.9" N 1°38`47.3"E (30) - 34°10`36.7" N 2°10`57.9"E (32)   n = 102 | - 34°27`43.21" N 2°10`09.20" E (1) - 34°19`06.89" N 1°58`48.29" E (2) - 34°21`36.73" N 1°58`33.04" E (3) - 34°32`18.70" N 2°16`33.02" E (2) - 34°22`46.96" N 2°19`14.03" E (1) - 34°20`48.27" N 2°18`58.84" E (3) - 33°54`24.81" N 2°33`32.28" E (1) - 33°45`24.13" N 3°01`48.98" E (2) - 33°48`15.19" N 3°11`31.91" E (3) - 34°10`28.53" N 3°00`25.09" E (3)   n = 21 |
| OULED-DJELLAL | DJELFA | - 35°36'36.20"N 3°20'8.12"E (45) - 35°30'15.82"N 2°53'15.37"E (31) - 35°20'41.08"N 2°28'33.39"E (33) - 35°25'32.88"N 2°29'11.70"E (40) - 35°25'23.81"N 2°19'35.57"E (38) - 35°29'26.38"N 3°10'18.79"E (30) - 34°47'42.52"N 2°56'48.40"E (45) - 34°47'12.43"N 2°50'50.57"E (35) - 34°51'56.64"N 2°51'36.53"E (36) - 34°52'25.55"N 2°53'11.75"E (31)   n = 364 | - 35°22'12.87"N 2°44'16.02"E (1) - 35°21'26.12"N 2°42'49.20"E (2) - 35°21'30.35"N 2°42'50.48"E (2) - 35°22'19.83"N 2°45'15.95"E (1) - 34°31'48.10"N 3°44'15.26"E (1) - 34°31'50.35"N 3°44'4.19"E (1) - 34°31'58.77"N 3°44'20.75"E (2) - 34°32'3.93"N 3°43'59.02"E (1) - 34°19'54.53"N 3°14'57.70"E (1) - 34°19'52.74"N 3°14'51.04"E (1) - 34°20'43.76"N 3°10'57.95"E (1) - 34°19'17.62"N 3° 7'47.94"E (2) - 35°36'36.20"N 3°20'8.12"E (2) - 35°29'26.38"N 3°10'18.79"E (3)   n = 21 |
|  | LAGHOUAT | - 33°45'50.20"N 3° 2'40.15"E (33) - 33°45'26.15"N 3° 2'40.50"E (40) - 33°34'17.36"N 2°54'28.03"E (36) - 33°30'14.98"N 2°54'10.39"E (39) - 33°21'21.86"N 3° 3'22.24"E (36) - 33°16'57.86"N 3° 0'9.95"E (36) - 33°49'56.57"N 3°11'1.12"E (39) - 33°49'56.02"N3°11'3.27"E (31) - 33°50'7.05"N 3°10'46.70"E (32) - 34°11'23.29"N 2°28'30.86"E (34)   n = 356 | - 33°49'57.00"N 3°11'3.00"E (2) - 33°49'55.00"N 3°11'3.00"E (3) - 33°45'52.50"N 3° 2'39.04"E (2) - 33°46'52.17"N 2°57'36.21"E (2) - 33°34'17.00"N 2°54'28.00"E (2) - 33°45'19.00"N 3° 2'13.00"E (2) - 33°16'60.00"N 3° 0'15.00"E (2) - 33°16'57.97"N 3° 0'14.80"E (1) - 33°16'56.62"N 3° 0'24.21"E (1) - 33°21'21.86"N 3° 3'22.24"E (3) - 33°47'12.50"N 2°57'7.89"E (1) - 33°53'42.55"N 2°32'1.81"E (1)   n = 22 |
|  | BISKRA | - 34°13'35.99"N 5° 4'13.79"E (34) - 34°14'30.52"N 5° 6'1.92"E (34) - 34°24'47.2"N 5° 2'7.2"E (31) - 34° 4'0.84"N 4°59'55.72"E (33) - 34° 3'14.40"N 5° 1'59.00"E (32) - 34° 3'49.41"N 5° 1'59.66"E (34)   n = 198 | - 34° 3'27.57"N 4°57'25.00"E (2) - 34° 3'16.00"N 5° 2'1.00"E (3) - 34° 3'11.50"N 5° 1'56.10"E (3) - 34°13'37.20"N 5° 4'18.60"E (3) - 34°14'26.70"N 5° 5'59.80"E (2) - 34°15'55.90"N 5°15'15.00"E (3) - 34°15'27.03"N 5°36'49.00"E (3) - 34°12'44.02"N 5°37'18.99"E (3) - 34°12'44.00"N 5°37'19.00"E (2) - 34°36'34.00"N 5°39'55.00"E (1) - 34°33'34.00"N 5°40'33.00"E (1) - 34°35'52.00"N 5°39'14.00"E (3)   n = 29 |
|  | M’SILA | - 36° 5'49.62"N 3°23'25.18"E (35) - 35°16'36.71"N 4°48'6.15"E (31) - 35°12'3.20"N 4°47'56.80"E (35) - 34°51'57.23"N 4°15'32.35"E (31) - 34°49'50.8"N 4°17'14.1"E (48)   n = 180 | - 34°48'51.93"N 4°11'39.84"E (2) - 34°48'37.28"N 4°10'51.56"E (2) - 34°51'16.27"N 4° 6'55.38"E (1) - 34°53'48.84"N 4°11'56.77"E (2) - 35°35'28.00"N 3°33'24.00"E (2) - 35°35'28.00"N 3°33'24.00"E (1) - 34°53'30.28"N 4° 9'55.67"E (2) - 35° 3'56.59"N 4°59'12.26"E (3) - 34°57'15.93"N 4°37'51.94"E (2) - 35° 2'34.91"N 4°38'20.15"E (2) - 35° 4'18.00"N 4°38'44.43"E (1)   n = 20 |

The number of individual considered in each place is indicated in brackets.
