# Supplementary material for: Assessing patterns of genetic admixture between sheep breeds: Case study in Algeria
Source: Ecol Evol. 2017 Jul 8;7(16):6404–12. doi: 10.1002/ece3.3069 (PMC5574784; doi:10.1002/ece3.3069)
Supplement: Supplementary file 2 [file ECE3-7-6404-s002.docx]

**Table S2.** Details concerning the diversity of the microsatellite markers considered.

| Locus Name  (Group of multiplex) | Chr. | PIC (%) | Na  (Na_e_) | MNA | Ho | H_e_ |
| --- | --- | --- | --- | --- | --- | --- |
| OarCP34 | 3 | 70.9 | 7  (4.0) | 7 | 0.73 | 0.75 |
| ILSTS11 | 9 | 59.1 | 8  (2.7) | 7 | 0.59 | 0.62 |
| INRA035 | 12 | 74.4 | 14  (4.5) | 11.5 | 0.79 | 0.78 |
| CSSM66 | 9 | 81.6 | 15  (6.0) | 13 | 0.70 | 0.83 |
| BM8125 | 17 | 66.6 | 11  (3.3) | 9.5 | 0.68 | 0.69 |
| DYMS1 | 20 | 86.1 | 14  (7.9) | 13 | 0.87 | 0.87 |
| MAF33 | 9 | 72.5 | 11  (4.2) | 10.5 | 0.79 | 0.76 |
| MCM140 | 6 | 82.6 | 16  (6.4) | 14 | 0.88 | 0.84 |
| OarFCB128 | 2 | 79.2 | 10  (5.4) | 9.5 | 0.79 | 0.82 |
| OarFCB193 | 11 | 82.5 | 15  (6.3) | 13.5 | 0.83 | 0.84 |
| MAF209 | 17 | 63.6 | 9  (3.0) | 8.5 | 0.60 | 0.67 |
| INRA063 | 14 | 85.9 | 18  (7.8) | 15 | 0.90 | 0.87 |
| OarJMP129 | 24 | 81.7 | 18  (6.1) | 14.5 | 0.86 | 0.83 |
| OarFCB304 | 19 | 81.3 | 14  (6.0) | 11.5 | 0.78 | 0.83 |
| MAF65 | 15 | 76.4 | 11  (4.8) | 10 | 0.42 | 0.79 |
| HUJ616 | 13 | 76.0 | 17  (4.7) | 15 | 0.44 | 0.79 |
| MAF70 | 4 | 87.0 | 18  (8.3) | 17.5 | 0.91 | 0.88 |
| OARJMP58 | 26 | 80.5 | 14  (5.7) | 13 | 0.80 | 0.82 |
| OARHH47 | 18 | 85.8 | 15  (7.7) | 14.5 | 0.88 | 0.87 |
| OARVH72 | 25 | 72.7 | 9  (4.0) | 9 | 0.78 | 0.75 |
| OARCB226 | 2 | 68.4 | 12  (3.3) | 11.5 | 0.73 | 0.70 |
| OarAE129 | 5 | 66.1 | 8  (3.3) | 7.5 | 0.75 | 0.70 |
| SRCRSP1 | CHI13 | 62.1 | 9  (3.1) | 7.5 | 0.66 | 0.67 |

Chr., chromosomal location; Na, Number of alleles; Na_e_, effective number of alleles; MNA, Mean Number of Alleles (considering Ouled-Djellal and Rembi breeds); H_e_, expected heterozygosity; H_o_, observed heterozygosity; PIC, Polymorphism Information Content.
